# Household Financial Burden and Poverty Impacts of Cancer Treatment in Vietnam

**DOI:** 10.1155/2017/9350147

**Published:** 2017-08-21

**Authors:** Van Minh Hoang, Cam Phuong Pham, Quynh Mai Vu, Thuy Trang Ngo, Dinh Ha Tran, Dieu Bui, Xuan Dung Pham, Dang Khoa Tran, Trong Khoa Mai

**Affiliations:** ^1^Hanoi University of Public Health, Hanoi, Vietnam; ^2^Nuclear Medicine and Oncology Center, Bach Mai Hospital, Hanoi, Vietnam; ^3^Vietnam National Cancer Hospital, Hanoi, Vietnam; ^4^Ho Chi Minh City Oncological Hospital, Ho Chi Minh City, Vietnam; ^5^Hanoi Oncology Hospital, Hanoi, Vietnam

## Abstract

**Purpose:**

This paper aims to analyze the household financial burden and poverty impacts of cancer treatment in Vietnam.

**Methods:**

Under the “ASEAN CosTs in ONcology” study design, three major specialized cancer hospitals were employed to assemble the Vietnamese data. Factors of socioeconomic, direct, and indirect costs of healthcare were collected prospectively through both individual interviews and hospital financial records.

**Results:**

The rates of catastrophic expenditure based on the cut-off points of 20%, 30%, 40%, and 50% of household's income were 82.6%, 73.7%, 64.7%, and 56.9%, respectively. 37.4% of the households with patient were impoverished by the treatment costs for cancer. The statistically significant correlates of the impoverishment problem were higher among older patients (40–60 years: 1.77, 95% CI 1.14–2.73; above 60 years: 1.75, 95% CI 1.03–2.98); poorer patients (less than 100% national income: 29, 95% CI 18.6–45.24; less than 200% national income: 2.89, 95% CI 1.69–4.93); patients who underwent surgery alone (receiving nonsurgery treatment: 2.46, 95% CI 1.32–4.59; receiving multiple treatments: 2.4, 95% CI 1.38–4.17).

**Conclusions:**

Lots of households were pushed into poverty due to their expenditure on cancer care; more actions are urgently needed to improve financial protection to the vulnerable groups.

## 1. Introduction

Cancer is known as a very severe disease in which malignant tumors and neoplasms develop uncontrollably and create serious harm to the human organs [1]. Cancer is found to be the leading cause of death worldwide. It was estimated that, in 2013, there were 14.9 million new cases of cancer and the number of deaths due to cancer was 8.2 million, which created a heavy burden of cancer worldwide (around 196.3 million DALYs) [2]. The economic impact of cancer is enormous for both the person with cancer and the society as a whole. The total economic impact of premature death and disability from cancers worldwide in 2008 was $895 billion. The three types of cancers that caused the most global economic impacts were lung cancer ($188 billion), colon/rectum cancer ($99 billion), and breast cancer ($88 billion) [3]. As the costs of treatment for cancer are usually substantial, many households and individuals with cancer are facing financial catastrophes or are even pushed into poverty because of the costs [4–6]. The household financial burden from chronic diseases impacted more on the poor and vulnerable populations [7]. Poor households are more likely to suffer disproportionately from the financial effects of the costs of treatment for cancer [6].

Like other developing countries, Vietnam is undergoing a rapid epidemiological transition resulting in an increase in chronic noncommunicable diseases, especially cancers [8]. According to Vietnam Ministry of Health, approximately 74.3 percent of the total disease burden in Vietnam was caused by noncommunicable diseases (NCDs) with cancer among the top ten causes. In Vietnam, it is estimated that about 150,000 people are newly diagnosed with cancer and more than 75,000 die of the disease every year. The costs of treating six common cancers, breast, ovary, liver, colon, stomach, and pharynx, accounted for 0.22 percent of the country's GDP in 2012 [9]. In Vietnam, household financial burdens caused by chronic diseases, including cancer, are now substantial. Households with NCD patients (including cancer cases) were 2.3 times more likely to be impoverished due to healthcare payment than other households [10]. Households that lived in slum areas and belonged to the poor or poorest socioeconomic groups were significantly associated with increased impoverishment because of healthcare spending on treatment for chronic diseases, including cancer [11].

Cancer prevention and control in Vietnam is still facing a number of challenges such as lack of comprehensive actions from involved stakeholders, unavailability of services for cancer screening and early detection at grassroots level of care, and shortage of human capacities and financial resources [9, 12]. Specifically, for example, even though tobacco control policies have been strengthened, the current cigarette excise tax in Vietnam is still low (only 65% of cigarette price before VAT or 41% of retailed price [13]) as compared to the level of at least 70% of retail price recommended by the World Health Organization [14] so its impacts on prevention of cigarette-related cancers is still limited. While the coverage of health insurance in Vietnam is now about 85%, the benefit package of health insurance scheme is not high. The insurance card holders have to be responsible for the remaining part of the costs (copayments) which are sometimes very high. In healthcare facilities, there are still no official regulations on cost containment, especially on the application of advanced medical technologies and expensive medicines. Fee-for-service is still the main provider payment method which tends to increase healthcare payment as well as financial burden on households in Vietnam [9].

Vietnam is now implementing the National Strategy for Cancer Control up to 2010 and 2020 with five main objectives: (1) reducing the incidence of tobacco-related cancers by 30%, compared to the year 2000; (2) ensuring HBV vaccination coverage for all newborns; (3) reducing breast, cervix, mouth, and rectum cancers mortality rates; (4) decreasing the proportion of advanced stage cancers from 80 to 50%; and (5) establishing a community-based terminal care system for cancer patients and ensuring sufficient supplies of essential drugs. One of the proposed strategies is to improve the use of scientific evidence in the planning, management, and policy-making process. In this context, more research on various aspects of cancer prevention and control is needed. While scientific evidence shows the rapid rise of the burden caused by cancers in Vietnam, little is known about the extent to which households in the country suffer from financial catastrophe or impoverishment caused by the disease. This paper aims to analyze the household financial burden and poverty impacts of cancer treatment in Vietnam.

## 2. Methods

### 2.1. Study Design and Study Subject

This is a facility-based study using prospective approach. This study was conducted as part of a regional study on the economic and health impact of cancer in eight countries in the ASEAN (Cambodia, Indonesia, Laos, Malaysia, Myanmar, the Philippines, Thailand, and Vietnam), the ASEAN CosTs in ONcology (ACTION) study [15]. This is a longitudinal cohort study conducted on a sample of 10,000 cancer patients. Patients diagnosed with cancer for the first time were consecutively recruited. Patients were interviewed at baseline (after diagnosis), three months, and 12 months. The primary outcome is incidence of financial catastrophe following treatment for cancer, defined as out-of-pocket healthcare expenditure at 12 months. Greater details of the study protocol can be found elsewhere [16].

### 2.2. Study Sites

Three major national level cancer centers in Vietnam were purposively chosen for this study, including Oncology Department of Bach Mai Hospital (located in the north, with more than 2000 staff and 1900 beds), Vietnam National Cancer Hospital (located in the north, with 800 staff and 1000 beds), and Ho Chi Minh City Oncological Hospital (located in the south, with more than 1000 staff and 1400 beds).

### 2.3. Data Collection

Data were collected from May 2012 to August 2014. Three data collecting periods were implemented including baseline and 3-month and 12-month follow-ups. Face-to-face interviews with cancer patients and their relatives were conducted by trained hospital nurses. The research tool was built by the ACTION Group, which was adjusted by the nation's context and was back translated to the local language. Data related to socioeconomic factors and direct and indirect expenditure on healthcare were collected from individual interviews; medical records were taken from the hospital system [16].

### 2.4. Variables and Definitions

To measure the financial catastrophe and impoverishment of household, the following definitions were applied during data analyses process.

#### 2.4.1. Out-of-Pocket Payments (OOP)

The term out of pocket referred to household spending at the point they received health services. These services include either inpatient services or outpatient services. Nonmedical spending such as transportation, food, or accommodation was also included into OOP payments. The reimbursements from health insurance were excluded from patients out of pocket.

#### 2.4.2. Catastrophic Expenditure

Catastrophic heath expenditure occurs when a household's total out-of-pocket health payments equal or exceed a certain level (20%, 30%, 40%, and 50%) of household's income (household's income could be understood as the household's capacity to pay) [17].

#### 2.4.3. Impoverishment

A nonpoor household is impoverished by healthcare payments when it becomes poor after paying for health services. Decision number 09/2011/QD-TTg issued by Vietnam Ministry of Labour, Invalid and Social Affairs on the norms for poor households was applied to classify poor and nonpoor households [18].

### 2.5. Data Analysis

Stata statistic software version 12 was used to analyze the data. Both descriptive and analytical statistics were performed. Logistic regressions were used to identify correlates of impoverishment due to healthcare payments. The dependent variable was dummy variable on impoverishment. Independent variables include gender, education, income, age, occupation, health insurance coverage, type of treatment (surgery alone, chemical or radiology or medicine alone, and combination of surgery and other treatments), grade of tumor, and type of cancer. A significance level of *p* < 0.05 was used.

### 2.6. Ethical Consideration

All three local institutional ethics committees approved the study. Information sheet was given to all invited participants and written consent was given by each participant to join the study. The study protocol was approved by the Ethical Review Board of the Vietnam Ministry of Health.

## 3. Results

### 3.1. General Characteristics of the Study Participants

During the study period, after exclusions due to patient or doctor refusals, 1,916 cancer patients were recruited into the study. The 3- and 12-month follow-up interviews were completed by 1,141 patients (59.6%) (patients were still alive and responsive to the survey). There were no significant differences in other sociodemographic, clinical, and economic characteristics of the recruited patients and those included in the analysis. Generally, the majority of participants were female (71%), from 44 to 60 years (53.3%), completed secondary or high school (58.6%), and did not have a paid work (63.2%). 74.9% of the study participants had health insurance. Most of them came from households with total annual income 200% higher than the basis of national income (41.5%). Breast cancer was the most common type of oncology (27.1%). 75.2% of screened tumors were graded as type III. Most patients received multiple treatments (56.5%).

### 3.2. Household Out-of-Pocket Payments for Cancer Treatment

Table 1 presents the total amount of money that household with cancer patient paid for healthcare services (OOP) during 12 months. The mean, median, and standard deviation of the OOP were 43.9, 33.4, and 51.3 million VND, respectively. The OOP were higher among patients (1) of male gender; (2) 44–60 years old; (3) with highest education level; (4) having paid work; (5) not having health insurance; (6) having income at 100%–200% of national level; (7) suffering from breast cancer; (8) having cancer stage II; and (9) receiving multiple treatments.

### 3.3. Patterns of Catastrophic Expenditure and Impoverishment

**Figure 1:**
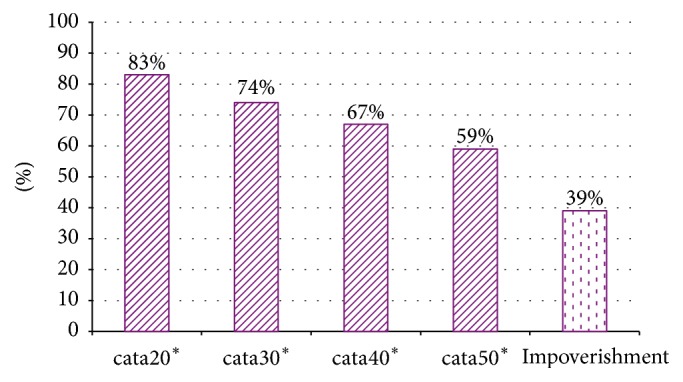
The rates of catastrophic expenditure and impoverishment among Vietnamese cancer patients. ^*∗*^Household had catastrophe expenditure if total medical expenditure equals or exceeds 20%/30%/40%/50% of total household income, respectively.

The rates of catastrophic expenditure and impoverishment are presented in Figure 1. The rates of catastrophic expenditure based on the cut-off points of 20%, 30%, 40%, and 50% of household's income were 82.6%, 73.7%, 64.7%, and 56.9%, respectively. 37.4% of the households with patient were impoverished by the treatment costs for cancer. Table 1 shows the patterns of catastrophic health expenditure and impoverishment by patient's characteristics.

The rates of financial hardship were higher among patients who (1) were 44–60 years old; (2) did not have health insurance; (3) suffered from breast cancer; (4) had cancer stage II; and (5) received multiple treatments.

### 3.4. Correlates of Impoverishment

Table 2 presents the results of logistic regression analysis of the correlates of impoverishment problem among the cancer patients. After controlling of confounding variables, the statistically significant correlates of the impoverishment problem were as follows.

#### 3.4.1. Age

The odds of being impoverished were higher among older patients as compared to the younger ones (OR for the group aged 44–60 years versus the group aged 44–60 years was 1.77; 95% CI: 1.14–2.73; OR for the group over 60 years versus the group 44–60 years was 1.75, 95% CI: 1.03–2.98).

#### 3.4.2. Income

The odds of being impoverished were higher among poorer patients as compared to the better offs (OR for the group with income below national income level versus the group with income 200% of national income level was 29, 95% CI: 18.6–45.24; OR for the group with income 100%–200% of national income level versus the group with income over 200% of national income level was 2.89, 95% CI: 1.69–4.93).

#### 3.4.3. Type of Treatments

Patients who underwent surgery alone had the lowest odds of being impoverished (OR for the patients who received chemotherapy or radiotherapy or hormonal therapy or biopharmaceutical therapy alone versus those who underwent surgery alone was 2.46, 95% CI: 1.32–4.59; OR for the patients who got multiple treatments versus those who underwent surgery alone was 2.4, 95% CI: 1.38–4.17).

## 4. Discussion

This research generates new evidence on household financial burden and poverty impacts of cancer treatment in Vietnam. The evidence is expected to be used in health planning, management, and policy-making process in the country and elsewhere. We have shown that a large proportion of Vietnamese households with cancer patient incurred catastrophic level of health expenditure and/or were pushed into poverty because of the costs of healthcare services (the rates of catastrophic expenditure based on the cut-off points of 20%, 30%, 40%, and 50% of household's income and impoverishment due to treatment costs for cancer were 82.6%, 73.7%, 64.7%, and 56.9%, resp.).

This finding is in line with other international studies which have proven the fact that household financial burden caused by cancer treatment is substantial. The ACTION study reported that, a year after diagnosis, only 23% of cancer patients from eight countries in ASEAN were alive with no financial catastrophe [15]. A study in Haiti found that two-thirds of women with breast cancer were to face financial catastrophe because of the treatment costs [19]. Similarly, cancer treatment is considered as the most costly healthcare service in India. Households with cancer patients spent 36–44% of their total annual expenditures and they might lose around 3% of the family workforce to spare time for patient care [20]. Another study from Pakistan also showed that the financial burden of cancer care was substantial and mostly borne by the patient or the family. Most of the time, the average monthly cost of treatment far exceeded the monthly household income [21]. Some recent literature reviews also indicated that households with chronic disease patients, including cancer patients, had to spend a substantial share of their incomes on care for these diseases and many households faced catastrophic health expenditure and impoverishment as a result of the spending [7, 22].

Our study revealed that the rates of catastrophic expenditure and impoverishment due to treatment costs for cancer were higher among older patients and those belonging to lower income families, having no paid work, not enrolled in health insurance scheme, and receiving multiple treatments methods. However, only older age, lower income, and receiving multiple treatment methods were shown to be statistically significant correlates of the impoverishment problem. At regional level, the ACTION study found that having a below-average income, having no health insurance, not having paid work, and having attended not higher than primary education were all associated with higher odds of experiencing catastrophic expenditure [15] and this reinforces the current knowledge of relationship between socioeconomically disadvantaged conditions and higher risks of financial hardship [23]. There should be financial support programs to cover the treatment costs of cancer and to help socioeconomically disadvantaged cancer patients to cope with the challenging situation.

Financial protection is the most important aspect of health insurance coverage but this research illustrates that, in Vietnam, health insurance had no statistically significant impacts on protecting households with cancer patients from impoverishment due to cancer treatment costs. This may be partially explained by the limitations of benefit packages of the health insurance programs. It fact, health insurance in Vietnam now covers part (0%–100%) of healthcare costs of the insured patients depending on the type of healthcare services. The insurance card holders have to be responsible for the remaining part of the costs (copayment). Many medicines and diagnostic tests for cancer are not covered by health insurance so the copayments are very high. Most of the studies on the impacts of health insurance in Vietnam consistently found that insurance has only a modest effect on reducing out-of-pocket payments [24–28]. Reform of health insurance benefit package to improve financial protection is needed in Vietnam. The ACTION study also found that, in the ASEAN region, the relationship between health insurance and financial catastrophe was not particularly strong [15].

The study has several limitations. Firstly, for pragmatic reasons, only hospitalized cancer patients were included and the findings may not be representative for the whole picture of household financial burden and poverty impacts of cancer treatment in Vietnam. Secondly, the low response rate (50%) may cause biases in the study finding. Thirdly, reporting income is regarded as a sensitive issue in Vietnam and the figures on income are normally underreported. Fourthly, only direct costs were included in this study. Sometimes, indirect costs (productivity loss and household suffer) are substantial and higher than the direct costs [6, 29]. Finally, the comparison of findings of this study with those from other contexts is just indicative because of the differences in definition and methods of calculation of catastrophic payment and impoverishment.

## 5. Conclusion

This study shows that a large proportion of Vietnamese households with cancer patient incurred catastrophic level of health expenditure and/or were pushed into poverty because of the costs of healthcare services. Socioeconomically disadvantaged cancer patients were particularly vulnerable to negative impacts of cancer treatment costs. Given the evidence, policy actions that can remove financial barriers and provide financial protection to the cancer patients as well as other groups of population are urgently needed. Cancer prevention strategies, especially effective tobacco control measures such as raising cigarette tax, would be prioritized actions in Vietnam. Other general interventions such as revision of health insurance package and reform of provider payment methods should be done as soon as possible. There should also be financial support programs to cover the treatment costs of cancer and to help socioeconomically disadvantaged cancer patients to cope with the challenging situation. A more representative study on household financial burden and poverty impacts of cancer treatment in Vietnam (using community-based approach or with larger sample size) is needed. We also need to include the indirect costs (opportunity costs) due to cancer treatment in the coming studies.

## Figures and Tables

**Table 1:** Patterns of household out-of-pocket payments and financial burdens for cancer treatments.

Subgroup	Total household out of pocket, million VND			Rate of catastrophe expenditure (using different cut-off points), %				Rate of impoverishment, %
	Mean	Median	SD	20%^*∗*^	30%^*∗*^	40%^*∗*^	50%^*∗*^	
*Gender*								
Male	46.206	33.65	66.772	79.8	71.6	65.6	56.8	33.5
Female	42.848	33.35	43.518	83.7	74.6	64.3	56.9	39
*Age group*								
<44	39.871	30	42.84	82.6	69.7	57.5	48.1	31.4
44–60	47.966	36.65	58.879	84.9	77.3	69.7	63.2	40.5
>60	38.058	30.39	37.504	76.8	69.5	60.6	51.6	37
*Education level*								
No school	40.688	25.6	69.24	82.1	76.9	66.7	59	41
Primary school	41.648	29.127	78.525	85.5	77	69.4	60.9	38.3
Secondary/high school	43.279	34	38.455	82.8	74.7	66.7	59.3	39.3
Vocational school	59.184	50.6	56.787	84.8	78.8	66.7	57.6	27.3
College/university	46.563	36.325	40.645	77	63	49.1	40.6	29.7
*Working status*								
Do not have paid work	42.404	31.27	56.289	81.4	73.6	65.5	58.9	38.4
Have paid work	46.232	37.2	41.442	84.5	73.8	63.3	53.3	35.7
*Health insurance status*								
Do not have health insurance	44.37	34.086	40.042	86.4	78.4	70.4	64.1	42.9
Have health insurance	43.634	33	54.586	81.3	72.1	62.8	54.4	35.6
*Household income level*								
<100% of mean national income	42.942	28.26	81.72	95.9	94.7	92.4	90.6	21.8
100%–200% of mean national income	46.1	38.5	38.738	96.7	92.3	89.2	83.3	73.3
>200% of mean national income	40.888	34.41	31.513	68.1	52.3	34.4	22.9	10.3
*Cancer site location*								
At hematological/blood system	28.533	19.925	23.966	59.1	50	40.9	27.3	13.6
At respiratory system	43.404	36	43.524	79.1	73.3	63.4	56	39.3
At digestive system	41.951	32.95	37.096	81.1	69.9	63.1	54.4	29.1
At reproductive system	40.558	32	37.037	84.9	76.8	68.1	58.4	37.8
Breast cancer	52.845	41.5	55.29	89.3	82.2	71.8	66.3	49.2
Other cancer	37.457	25	69.969	78.1	65.8	57	48.7	29.4
*Cancer grade*								
Cancer grade I	42.577	31.5	35.513	84	74.1	65.4	59.3	37
Cancer grade II	52.464	37.56	66.386	89.6	83.2	72.8	64.9	43.6
Cancer grade III	41.823	33	48.098	80.8	71.4	62.7	54.8	36
*Type of treatment*								
Surgery alone	28.236	20	40.612	76.5	62.7	53.6	43.1	25.5
Nonsurgery treatments^*∗∗*^	43.347	35	63.233	78.6	70.5	62.4	55.8	35.3
Multiple treatments^*∗∗∗*^				86.1	78	68.5	60.7	41.4

*Overall (n, %)*	*43.818*	*33.35*	*51.322*	*848 (82.6)*	*766 (73.7)*	*701 (64.7)*	*616(56.9)*	*406 (37.4)*

^*∗*^Catastrophe expenditure if total medical expenditures equal or exceed 20%/30%/40%/50% of total household income, respectively. ^*∗∗*^Nonsurgery treatments include chemotherapy or radiotherapy or hormonal therapy or biopharmaceutical therapy alone. ^*∗∗∗*^Multiple treatments include both surgery and nonsurgery treatments.

**Table 2:** Logistic regression analysis of the correlates of impoverishment.

Subgroup	Odd ratio	95% CI	
		Lower bound	Upper bound
*Gender*			
Male	Ref		
Female	1.03	0.64	1.65
*Age group*			
<44	Ref		
44–60	1.77^*∗*^	1.14	2.73
>60	1.75^*∗*^	1.03	2.98
*Education level*			
No school	Ref		
Primary school	0.73	0.27	1.93
Secondary/high school	0.88	0.34	2.24
Vocational school	1.05	0.25	4.37
College/university	1.22	0.42	3.51
*Working status*			
Do not have paid work	Ref		
Have paid work	1.16	0.79	1.71
*Health insurance status*			
Do not have health insurance	Ref		
Have health insurance	0.99	0.66	1.5
*Household income level*			
<100% of mean national income	29.0^*∗*^	18.6	45.24
100%–200% of mean national income	2.89^*∗*^	1.69	4.93
>200% of mean national income	Ref		
*Cancer site location*			
At hematological/blood system	Ref		
At respiratory system	1.76	0.34	9.07
At digestive system	1.39	0.27	7.22
At reproductive system	1.89	0.36	9.99
Breast cancer	2.19	0.42	11.42
Other cancer	1.71	0.33	8.78
*Cancer grade*			
Cancer grade I	Ref		
Cancer grade II	0.6	0.26	1.39
Cancer grade III	0.77	0.36	1.64
*Type of treatment*			
Surgery alone	Ref		
Nonsurgery treatments^*∗∗*^	2.46^*∗*^	1.32	4.59
Multiple treatments^*∗∗∗*^	2.40^*∗*^	1.38	4.17

^*∗*^Statistically significant result. ^*∗∗*^Chemotherapy or radiotherapy or hormonal therapy or biopharmaceutical therapy alone. ^*∗∗∗*^Multiple treatments include both surgery and nonsurgery treatments.
